# Age-related vulnerability of the human brain connectome

**DOI:** 10.1038/s41380-023-02157-1

**Published:** 2023-07-06

**Authors:** Massimo Filippi, Camilla Cividini, Silvia Basaia, Edoardo G. Spinelli, Veronica Castelnovo, Michela Leocadi, Elisa Canu, Federica Agosta

**Affiliations:** 1 Neuroimaging Research Unit, Division of Neuroscience, IRCCS San Raffaele Scientific Institute, Milan, Italy; 2 Neurology Unit, IRCCS San Raffaele Scientific Institute, Milan, Italy; 3 Neurophysiology Service, IRCCS San Raffaele Scientific Institute, Milan, Italy; 4 Neurorehabilitation Unit, IRCCS San Raffaele Scientific Institute, Milan, Italy; 5 Vita-Salute San Raffaele University, Milan, Italy

**Keywords:** Predictive markers, Neuroscience

## Abstract

Multifactorial models integrating brain variables at multiple scales are warranted to investigate aging and its relationship with neurodegeneration. Our aim was to evaluate how aging affects functional connectivity of pivotal regions of the human brain connectome (i.e., hubs), which represent potential vulnerability ‘stations’ to aging, and whether such effects influence the functional and structural changes of the whole brain. We combined the information of the functional connectome vulnerability, studied through an innovative graph-analysis approach (stepwise functional connectivity), with brain cortical thinning in aging. Using data from 128 cognitively normal participants (aged 20–85 years), we firstly investigated the topological functional network organization in the optimal healthy condition (i.e., young adults) and observed that fronto-temporo-parietal hubs showed a highly direct functional connectivity with themselves and among each other, while occipital hubs showed a direct functional connectivity within occipital regions and sensorimotor areas. Subsequently, we modeled cortical thickness changes over lifespan, revealing that fronto-temporo-parietal hubs were among the brain regions that changed the most, whereas occipital hubs showed a quite spared cortical thickness across ages. Finally, we found that cortical regions highly functionally linked to the fronto-temporo-parietal hubs in healthy adults were characterized by the greatest cortical thinning along the lifespan, demonstrating that the topology and geometry of hub functional connectome govern the region-specific structural alterations of the brain regions.

## Introduction

Aging is a complex biological process that implies brain morphological and functional changes associated with the accumulation of molecular and cellular damage over time [1].

Magnetic resonance imaging (MRI) has become a powerful, non-invasive tool to explore in vivo neuroanatomical alterations in the aging brain [2]. Overall, age-related morphometric changes are widespread across the cortex, although there are specific regions, as the frontal, parietal and temporal cortices characterized by a more prominent cortical thinning [3–7]. On the other hand, the effect of age also reflects on the functional brain architecture [2]. Functional signature of aging is a decreased co-activation of the default mode network (DMN), which finds general agreement across studies [2, 8–11], while age-related alterations have been observed less consistently in the salience, dorsal attention and sensorimotor networks [9, 12, 13].

In the recent years, the application of connectomics approaches has allowed to investigate age-related alterations in the functional connectivity between networks in addition to those within networks, by evaluating segregation and integration properties [14, 15]. There is a convergent observation that aging leads to lower within- and higher between-network connectivity, which might reflect in an imbalance towards increasing integration at the expense of segregation [16–18]. Moreover, in line with such results local efficiency and modularity, strictly related to segregation property, are typically decreased in older relative to younger adults, whereas global efficiency, strictly related to integration, is quite preserved [19–22].

The underpinning assumption of these changes in brain efficiency relies on the age-related alterations of highly connected pivotal regions of the neurocognitive functional networks, namely brain hubs. Indeed, across lifespan, a functional rearrangement of these regions has been shown, characterized by an exchange of high-degree hubs to low-degree nonhub regions reflecting compensatory or over-recruitment mechanisms to meet the challenges of functional decline [18, 19]. The high neural and metabolic activity of hubs across lifespan [23] makes these regions more vulnerable to the accumulation of biological damage at the extent that spatial convergence has been shown between cortical hubs and alterations in structural integrity, dopaminergic dysfunction and non-pathological amyloid-β deposition [11, 24–26]. In support of this, a co-localization of alterations in functional connectivity and atrophy in clinically normal elderly individuals has been observed [27]. However, the cause-effect relationship according to which alterations in the topography of brain functional connectivity architecture might govern region-specific structural changes, although hypothesized in the context of neurodegenerative pathologies [28–32], has not been demonstrated yet with aging.

By translating this assumption to aging, we suggest that functional network rearrangements of brain hubs and their structural change trajectories across lifespan influence the functional and structural trend of changes of the remaining brain regions (Supplementary Figure S1). To verify such hypothesis, we proposed a model that integrates the information of the functional connectome vulnerability, studied through an innovative graph-analysis approach (i.e., stepwise functional connectivity [SFC]), with brain cortical thinning in aging.

## Patients and methods

### Participants

One hundred twenty-eight healthy subjects were recruited by word of mouth at the IRCCS San Raffaele Scientific Institute (Milan, Italy) from 2017 to date in the framework of an observational study. None of the participants had any history of psychiatric or neurological disorder, drug or alcohol abuse, or any systemic disease that might compromise cognitive function or blood flow (e.g., diabetes, untreated hypertension, cardiovascular disease). All participants scored in the normal range on the Mini Mental Status Exam (≥27) [33]. Prior to participation, written informed consent was obtained from all subjects. Participants aged 20–85 years and were divided into two groups according to age: 50 young adults (≤35 years old) and 78 older adults (>35 years old). At study entry, both groups performed a comprehensive neuropsychological and behavioral evaluation (Supplementary Table S1 in Supplementary Information) and underwent brain MRI scan, including 3D high resolution T1-weighted sequence and T2* weighted (GE-EPI) as resting-state functional sequence (Supplementary Table S2 in Supplementary Information).

### Statistical analysis: demographic, clinical and cognitive data

Demographic and cognitive data were compared between the two groups using one-way ANOVA models—for continuous variables - or Chi-square test - for categorical variables (Table S1 in Supplementary Information). Cognitive data analysis was corrected for age, sex, and education. Two-sided p-value < 0.05 was considered for statistical significance. *P* values were adjusted for Bonferroni multiple comparisons. Analyses were performed using R Statistical Software (version 4.0.3; R Foundation for Statistical Computing, Vienna, Austria).

### MRI analysis

MRI data were analyzed at the Neuroimaging Research Unit, Division of Neuroscience, IRCCS San Raffaele Scientific Institute and Vita-Salute San Raffaele University, Milan, Italy. The study framework consisted of (i) identifying highly functionally connected brain regions with selected hubs in young subjects; (ii) comparing hub functional connectivity maps between young and old adults; (iii) modeling cortical thickness trajectories across lifespan and finally, (iv) investigating the relationship between functional connectivity patterns in young subjects and cortical thickness loss of highly hub-connected regions in old subjects.

#### (i) Identification of brain regions highly functionally connected with hubs in healthy young subjects

We performed SFC analysis, a novel graph-theory-based method, characterizing functional connectivity between a priori seed of interest and other brain regions at different steps [34, 35]. A step refers to the number of links (edges) that belongs to a path connecting a node (brain cortical region) to the seed area. The pipeline adopted for this study, involving RS-fMRI pre-processing, functional connectome reconstruction and SFC implementation has been recently described [32]. We reconstructed maps up to four steps, since it has been previously showed that SFC patterns become stable for link-step distances above four [32]. We will refer to functional connectivity at one-link step as direct connectivity with the seed and to functional connectivity at subsequent steps (2–4) as indirect connectivity [34, 35]. The a priori selected hubs of the healthy human brain were middle frontal gyrus, rostral anterior cingulate cortex, inferior parietal cortex, precuneus, posterior cingulate cortex, middle temporal gyrus, lingual and pericalcarine cortex [36–38]. Hereafter, we will refer the fronto-temporo-parietal hubs as FTP hubs and the occipital hubs as OCC hubs. We calculated the average SFC maps for each step in the young group.

To identify highly connected regions with the hubs, average SFC maps for each of the four steps were obtained averaging all the young healthy subject maps. The SFC maps were then projected onto the cerebral hemispheres of the Population-Average Landmark and Surface-based (PALS) surface (PALS-B12) provided with Caret software using the “enclosing voxel algorithm” and “multifiducial mapping” settings.

#### (ii) Stepwise functional connectivity alterations among hubs themselves and highly connected regions in old relative to young healthy subjects

To identify regions that demonstrated between-group differences in stepwise connectivity, SFC maps across different link-step distances (i.e., SFC maps 1 to 4) were compared between young and older adults. Voxel-wise analyses were performed using general linear models as implemented in SPM12. Whole-brain two-sample t-test comparisons between groups were performed, including age, sex, and education as covariates. A threshold-free cluster enhancement method, combined with nonparametric permutation testing (5000 permutations) as implemented in the Computational Anatomy Toolbox 12 (CAT12, http://www.neuro.uni-jena.de/cat/) was used to detect statistically significant differences at *p* < 0.05, family-wise error (FWE) corrected. Such analyses allowed the identification of specific regions that demonstrated between-group differences in stepwise connectivity. As a final step, all resulting maps from statistical analysis were projected onto the cerebral hemispheres of the PALS surface, as for average SFC maps. Furthermore, to identify regions that demonstrated between-gender differences, SFC maps across different link-step distances (i.e., SFC maps 1 to 4) were compared between men and women in older adults.

#### (iii) Modeling of cortical thickness trajectories with aging

Regional cortical thickness was estimated in all subjects on 3D T1-weighted images using FreeSurfer (version 5.3) image analysis suite (http://surfer.nmr.mgh.harvard.edu/). Briefly the WM/GM boundary was automatically delineated following the intensity gradients. The cerebral cortex was parcellated into 68 cortical regions based on Desikan atlas and mean cortical thickness was calculated per each region as the average shortest distance between WM borders and pial surface. Subcortical regions were not included in such analysis due to the low accuracy of such framework for deep cerebral structures.

The relationship between individuals’ age and cortical thickness of each cortical region was modeled using Gaussian Process Regression (GPR), a nonparametric Bayesian approach able to reconstruct evolution trajectories by interpolation with the observed data [39–42]. Per each cortical region, we obtained predicted age cortical trajectories ranging from 20.5 to 84.6 years, including sex and education as covariates. To identify the regional cortical thinning, the relative change across lifespan was assessed by performing rank transformation on cortical regions based on their thickness values at either the ends of the observed timeframe (20.5 and 84.6 years). Regions that showed to be thinner compared to other cortical regions were attributed to a higher rank. Then, we evaluated the rank variation per each region by subtracting the starting rank value (at 20.5 year) of each cortical region to its final rank value (at 84.6 years). As such, positive values of change reflected regions with the highest change rate across lifespan, while negative values present regions with less change. Based on such rank variation, cortical regions were ordered by the region that varies the least to that which varies the most. Finally, rank variation was Z-scored and a correlation was performed between the regional cortical mean thickness values and the degree of thickness variation along lifespan, to evaluate whether there was a relation between the pattern of changes and the brain pattern of cortical thickness across lifespan. The same analysis has been reported separately for men and women. We obtained also differences of the cortical thickness regional change in men and women between 20.5 (starting rank value) and 84.6 years (final rank value) of age.

#### (iv) Cortical thickness loss of highly connected regions

We investigated the spatial similarity between young SFC pattern and cortical atrophy in old subjects. The hypothesis is that brain regions highly connected with hubs in the young healthy brain are those that change with aging and become atrophic first.

In the young adult group, per each seed region, the combined version of SFC maps from 1 to 4 was computed into one single map (combined SFC maps), whose values ranged from 1 to 4 steps (1=closer to the seed; 4=far from the seed). We identified the highest functional connectivity of each voxel among the four values of each map and set the values of each voxel with the number of steps in which the functional connectivity resulted maximized. The mean combined SFC map of young group was obtained, by averaging all the young subject maps. Then, regional cortical thickness values per each old adult were normalized relative to the mean and the standard deviation of regional values of young adult group and, subsequently, a group average was computed per each region.

Correlation analysis between the combined SFC maps of young subjects and mean cortical thickness values of old subjects was performed. Moreover, the distribution of cortical thickness values of those regions functionally closest to each seed (SFC values < 1.5) within older group was visually reported. Finally, cortical thickness values of these regions were compared between the two groups using ANOVA models, Bonferroni-corrected for multiple comparisons at level of 0.05, adjusted for sex and education.

## Results

### (i) Identification of brain regions highly functionally connected with hubs in healthy young subjects

**Fig. 1:**
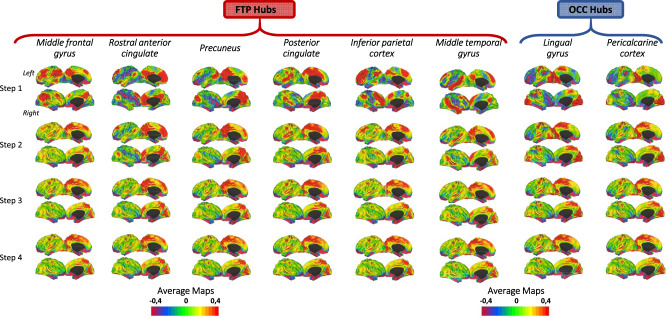
Stepwise functional connectivity average maps in young healthy adults. Cortical maps represent characterization of stepwise connectivity analysis from the FTP and the OCC hubs in healthy young adults. Results are depicted in surface space per each of the eight hubs. Yellow/red areas represent strong functional connectivity with the hub, whereas blue/violet areas represent weak functional connectivity. FTP fronto-temporo-parietal, OCC occipital.

Investigating the average SFC maps from each hub (i.e., seeds) in young adults, we found that all seed regions exhibited a dense and predominant regional-local direct functional connectivity (Fig. 1). FTP hubs revealed a common pattern of direct connections, reaching firstly superior frontal gyrus, supramarginal gyrus, superior and inferior parietal, isthmus and posterior cingulate and precuneus. On the other hand, OCC hubs shared a pattern of dense direct connections within occipital lobe (lateral occipital gyrus, cuneus, lingual, pericalcarine) and the sensorimotor areas (precentral, postcentral and paracentral gyri). Of note, the precuneus hub is the only seed region directly connected both to OCC and FTP hubs (Fig. 1).

Looking at the indirect connectivity (intermediate steps), FTP hubs revealed a common pattern reaching occipital regions (cuneus, lingual and fusiform), superior temporal and sensorimotor areas (paracentral, precentral and postcentral gyri) more prominently in the third and the fourth step. The OCC hubs indirectly connected to the superior frontal, caudal anterior cingulate and superior temporal areas (Fig. 1).

### (ii) Functional connectivity alterations among hubs themselves and highly connected regions in old relative to young healthy subjects

**Fig. 2:**
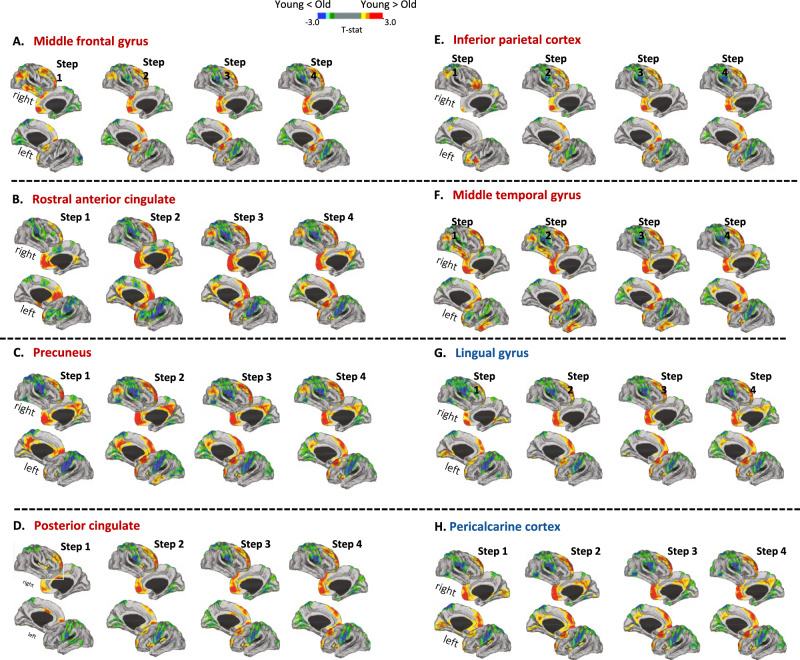
Differences between young and old healthy adults in stepwise functional connectivity of the eight hubs. **A**–**H** Cortical maps represent the significant differences in stepwise functional connectivity values between young healthy adults and older healthy adults. Statistical analysis was adjusted for sex and education. Results were corrected for multiple comparisons using a threshold-free cluster enhancement method combined with nonparametric permutation testing at *p* < 0.05 FWE-corrected. Color bars show the t-statistic applicable to the image. Red-yellow areas represent decreased functional connectivity in older adults relative to young adults, whereas blue/green areas represent enhanced functional connectivity in older adults compared to young.

When comparing SFC maps of FTP hubs between the two groups, older adults showed decreased direct functional connectivity in the superior frontal gyrus, medial orbitofrontal cortex and inferior parietal relative to young adults (Fig. 2A–F—red/yellow areas in step 1). Reduced indirect functional connectivity was found in frontal lobe (superior frontal gyri, medial orbitofrontal cortex and caudal anterior cingulate), parietal lobe (precuneus, posterior cingulate and inferior parietal cortex), and insula (Fig. 2A–F—red/yellow areas in steps 2–4). Regarding the opposite contrast, older group exhibited enhanced direct functional connectivity from FTP hubs to sensorimotor cortex (precentral and postcentral gyri), superior parietal cortex, supramarginal and superior temporal gyri (Fig. 2A–F—blue/green areas in step 1). Moreover, older subjects showed additional enhanced indirect functional connectivity within occipital lobe, specifically in lingual gyrus, pericalcarine, cuneus, fusiform gyrus (Fig. 2A–F—blue/green areas in steps 2–4).

Regarding direct functional connectivity from the OCC hubs, older adults showed decreased functional connectivity relative to the young group in frontal lobe (superior frontal gyri and medial orbitofrontal cortex), isthumus cingulate cortex and lingual gyri (Fig. 2G, H—red/yellow areas in step 1). Moreover, older group were characterized by decreased indirect connectivity within frontal lobe (superior frontal gyri, medial orbitofrontal cortex and rostral anterior cingulate), parietal lobe (precuneus and posterior cingulate) and insula (Fig. 2G, H—red/yellow areas in steps 2–4). Referring to the opposite contrast, older healthy adults showed increased direct functional connectivity relative to young subjects within the sensorimotor cortex (precentral and postcentral gyri), parietal lobe (superior parietal cortex and supramarginal gyrus) and the superior temporal gyrus (Fig. 2G, H blue/green areas in step 1). Regarding indirect connectivity, older subjects were characterized by increased functional connectivity in fusiform gyrus and occipital lobe (lingual gyri, pericalcarine and cuneus) (Fig. 2G, H—blue/green areas in steps 2–4).

Regional differences between older and young adults per each SFC map are reported in detail in Table S3 in Supplementary Information.

When comparing SFC maps of FTP hubs between men and women within the older adults group, men showed decreased direct and indirect functional connectivity in the superior frontal gyrus, caudal anterior and posterior cingulate gyrus and middle/inferior temporal gyri, relative to women (Supplementary Figure S2A-F – red/yellow areas in steps 1–4). On the contrary, men compared with women exhibited enhanced direct and indirect functional connectivity from FTP hubs to sensorimotor cortex (precentral and postcentral gyri) and supramarginal gyri (Supplementary Fig. S2A–F—blue/green areas in steps 1–4). Regarding direct and indirect functional connectivity from the OCC hubs, men showed decreased functional connectivity relative to women in the caudal anterior and posterior cingulate gyri, middle/inferior temporal and lingual gyri (Supplementary Fig. S2G, H—red/yellow areas in steps 1–4). In addition, men exhibited enhanced direct and indirect functional connectivity from OCC hubs to sensorimotor cortex (precentral and postcentral gyri) and supramarginal gyri (Supplementary Fig. S2G, H—blue/green areas in steps 1–4).

### (iii) Modeling of cortical thickness trajectories with aging

We modeled the regional change in cortical thickness on the entire cohort (*n* = 128) between 20.5 and 84.6 years of age. The 97% of cortical regions showed decreasing thickness with advancing age (Supplementary Fig. S3A). Calculating the relative change across lifespan, through the rank transformation, the highest cortical thinning was observed in the majority of FTP hubs (right middle frontal gyrus, rostral anterior cingulate, precuneus, right posterior cingulate, inferior parietal cortex and middle temporal gyrus) and in regions belonging to the temporal lobe (parahippocampal, superior temporal gyrus, transverse temporal gyrus and fusiform), frontal lobe (lateral orbitofrontal, superior and inferior frontal including pars triangularis, pars opercularis), parietal lobe (the isthmus of cingulate and supramarginal) and insular cortex (Supplementary Fig. S3B and Table 1). On the other hand, OCC hubs, as well as other occipital regions (cuneus and lateral occipital) and the sensorimotor and premotor areas (precentral, postcentral and paracentral regions), showed the lowest cortical thickness change in relation to the whole brain across lifespan (Supplementary Fig. S3B and Table 1). Investigating between-gender differences, men and women were characterized by decreased cortical thickness in all the brain lobes during aging, but with pronounced degeneration in men compared to women in frontal and temporal regions (Supplementary Fig. S4). Particularly, 71% of the brain regions (including all the frontal and temporal regions) showed greater changes in men than women.

**Table 1:** Rank of the cortical regions based on their cortical thinning across lifespan.

Desikan regions	Final rank	Desikan regions	Final rank
L parahippocampal	66	L inferior parietal	33
R pars triangularis	65	R rostral anterior cingulate	32
R superior temporal	64	R lateral occipital	31
R middle temporal	63	L pars triangularis	30
R superior frontal	62	L lateral occipital	29
L middle temporal	61	R precentral	28
L superior temporal	60	R pars orbitalis	27
R pars opercularis	59	L lateral orbitofrontal	26
L transverse temporal	58	L lingual	25
R parahippocampal	57	L precentral	24
R precuneus	56	R inferior temporal	23
R insula	55	L medial orbitofrontal	22
R bankssts	54	L frontal pole	21
L fusiform	53	L posterior cingulate	20
L pars opercularis	52	R paracentral	19
L isthmus cingulate	51	R lingual	18
R isthmus cingulate	50	L superior parietal	17
R supramarginal	49	L postcentral	16
L bankssts	48	R entorhinal	15
R posterior cingulate	47	R postcentral	14
R fusiform	46	L inferior temporal	13
L insula	45	R superior parietal	12
L temporal pole	44	L paracentral	11
L supramarginal	43	R temporal pole	10
R transverse temporal	42	L cuneus	9
L precuneus	41	R medial orbitofrontal	8
L superior frontal	40	R caudal anterior cingulate	7
L pars orbitalis	39	L middle frontal	6
L entorhinal	38	R cuneus	5
R middle frontal	37	R frontal pole	4
R lateral orbitofrontal	36	L pericalcarine	3
L rostral anterior cingulate	35	R pericalcarine	2
R inferior parietal	34	L caudal anterior cingulate	1

Considered cortical regions were ordered by the region that varies the least (position 1) to that which varies the most (position 66), based on rank variation of each brain region. The rank variation has been calculated as the difference between the rank position at the end of the observed timeframe and the rank position at the beginning of the observed timeframe.

*L* left, *R* right.

Finally, we observed a positive relationship (r = 0.32, *p* = 0.01) between the regional mean thickness values and their changes across lifespan (Supplementary Fig. S3C), suggesting that regions with greater cortical thickness are those that change more in lifespan.

### (iv) Cortical thickness loss of highly connected regions

**Fig. 3:**
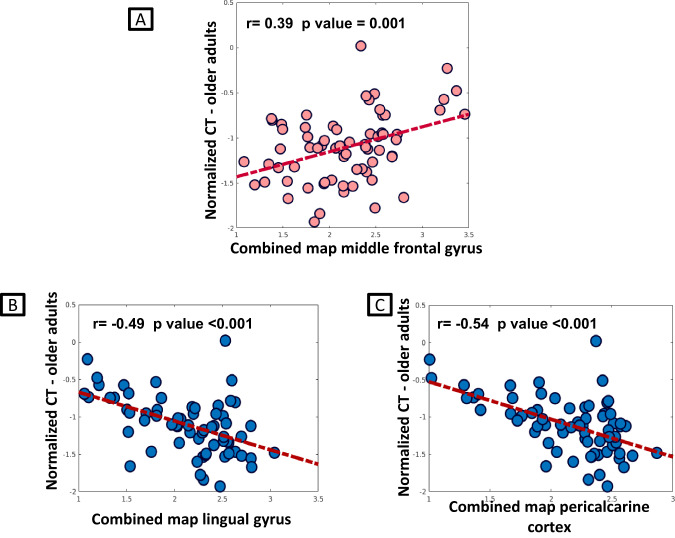
Correlation analysis between combined SFC maps of young group and normalized cortical thickness values in older adults. Relationship between combined SFC maps of the middle frontal gyrus (FTP hub) (**A**) and OCC hubs (**B**, **C**) and the cortical thickness of older adults normalized relative to young adults. FTP fronto-temporo-parietal, OCC occipital, SFC stepwise functional connectivity.

From correlation analysis between combined SFC maps of hubs of the young group and mean regional cortical thickness values of older adults, significant positive correlation between SFC pattern of middle frontal hub in young adults and mean cortical thinning in older adults was found (Fig. 3A). Whereas significant negative correlation emerged between the SFC pattern of lingual and pericalcarine hubs and mean cortical thinning in older adults (Fig. 3B, C).

**Fig. 4:**
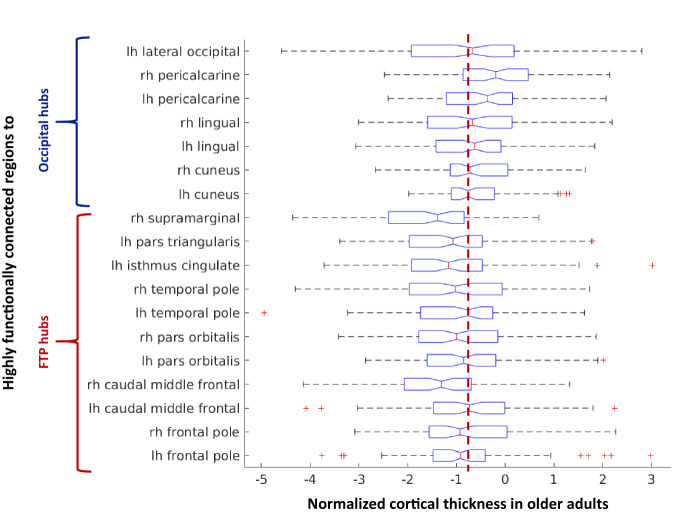
Boxplot of normalized cortical thickness values of highly connected regions with hubs in older healthy adults. Cortical thickness value distribution of brain regions highly functionally connected to FTP hubs and OCC hubs. The red dotted line qualitatively highlights that cortical thickness values of the FTP-linked regions are lower than those of OCC-linked regions. FTP fronto-temporo-parietal, OCC occipital.

Then, the functionally closest regions to FTP hubs were frontal pole, caudal middle frontal, pars orbitalis and temporal pole bilaterally and left isthmus cingulate, left pars triangularis and right supramarginal (FTP-linked regions). The functionally closest regions to OCC hubs were instead the hubs themselves (lingual gyrus and pericalcarine cortex) and cuneus (OCC-linked regions). Comparing mean cortical thickness values of FTP- and OCC-linked regions between the two groups, older adults showed significant reductions relative to young adults (Table 2 and Fig. 4). Finally, investigating the distribution of normalized cortical thickness values in older adults, FTP-linked regions showed a greater mean cortical thinning than OCC-linked regions (Fig. 4).

**Table 2:** Cortical thickness values of brain regions highly functionally connected to FTP and OCC hubs in young and older healthy adults.

Highly functionally connected regions to	Young healthy adults	Older healthy adults	*p* value
Fronto-temporo-parietal hubs			
R frontal pole	2.91 ± 0.27	2.71 ± 0.27	<0.001
L frontal pole	2.88 ± 0.20	2.70 ± 0.24	0.001
R caudal middle frontal	2.61 ± 0.11	2.46 ± 0.12	<0.001
L caudal middle frontal	2.56 ± 0.12	2.46 ± 0.14	0.02
R pars orbitalis	2.79 ± 0.18	2.62 ± 0.18	<0.001
L pars orbitalis	2.84 ± 0.21	2.66 ± 0.20	<0.001
R temporal pole	4.05 ± 0.20	3.84 ± 0.26	0.001
L temporal pole	3.99 ± 0.23	3.78 ± 0.28	0.002
L isthmus cingulate	2.55 ± 0.17	2.36 ± 0.21	<0.001
L pars triangularis	2.52 ± 0.15	2.35 ± 0.17	<0.001
R supramarginal	2.66 ± 0.11	2.49 ± 0.12	<0.001
Occipital hubs			
R cuneus	1.83 ± 0.14	1.75 ± 0.13	0.01
L cuneus	1.86 ± 0.17	1.76 ± 0.13	0.01
R lingual	2.00 ± 0.11	1.92 ± 0.13	0.01
L lingual	2.00 ± 0.13	1.91 ± 0.13	0.001
R pericalcarine	1.48 ± 0.13	1.45 ± 0.13	0.04
L pericalcarine	1.52 ± 0.14	1.45 ± 0.13	0.003
L lateral occipital	2.25 ± 0.10	2.18 ± 0.15	0.13

Values are reported in millimeters and represent the means ± standard deviation. *P* values referred to analysis of variance models, followed by post-hoc pairwise comparisons (Bonferroni-corrected for multiple comparisons). Education and sex were included as covariates.

*L* left, *R* right.

## Discussion

The study aim was to evaluate how aging affects functional connectivity of the human brain connectome hubs, which represent potential vulnerability ‘stations’ to aging [18], and how such effects influence the vulnerability and structural changes of the whole brain. Overall, studying the topological functional network organization in the optimal healthy condition (i.e., young cognitively unimpaired adults), we found that FTP hubs showed a highly direct functional connectivity with themselves and among each other, while OCC hubs showed a direct functional connectivity within occipital regions and sensorimotor areas. Modeling cortical thickness changes over lifespan, we observed that FTP hubs were among the brain regions that changed the most. On the contrary, OCC hubs showed a quite spared cortical thickness across ages. Finally, we demonstrated that cortical regions highly functionally linked to the FTP hubs in healthy adults were characterized by the greatest cortical thinning along the lifespan.

In young adults, the functional organization starting from FTP hubs resulted densely and directly connected: (i) with themselves; (ii) among FTP hubs (inferior parietal gyrus, posterior cingulate cortex and precuneus); and (iii) with superior frontal gyrus, isthmus cingulate cortex, supramarginal gyrus and superior parietal gyrus. These results are quite expected and confirm the current knowledge [35, 43]. It is important to note that regions connected with FTP hubs tend to resemble the classic DMN [44], salience and executive control networks, highlighting the rich interplay among such functional circuits [35, 43]. On the other hand, we observed that OCC hubs were densely and directly connected with the occipital regions, sensorimotor regions (precentral, postcentral and paracentral gyri) and precuneus, in line with recent studies suggesting an early integration of somatomotor and visual processing for oculomotor functions [35]. In addition, the precuneus appeared especially central in linking the other FTP hubs and the OCC hubs, serving as functional communication bridge, confirming its role of connector hub [38, 45, 46]. Focusing on the indirect connections (intermediate step-link), all the selected cortical hubs reached the remaining regions, shaping a whole-brain stable map of functional connectivity, supporting the global functional integration properties of the healthy brain [47].

With increasing age, we observed that its effect on such functional network organization translates into functional alterations primarily in the hubs themselves and the regions directly connected to them, and then in the indirectly connected regions, following the topological functional network organization identified in the healthy young subjects. Indeed, we found significantly decreased functional connectivity with aging within both FTP and OCC hubs [9], and also in those regions directly connected to FTP hubs and mainly distributed in the fronto-temporo-parietal areas. These results are consistent with previous studies, suggesting the loss of segregation properties and a functional decline of high-degree hubs with aging [16–18]. Moreover, our findings highlighted a greater vulnerability to aging of FTP hubs and fronto-temporo-parietal regions in general [2, 19], which, in addition, are known to be affected in many neurodegenerative disorders [23, 48–50].

Importantly, hyperconnectivity was also observed when aging occurred, particularly in precuneus, superior parietal lobule and sensorimotor network, both in FTP and OCC hub maps. Such hyperconnectivity among regions belonging to different networks could be interpreted as the attempt to compensate for the functional decline within networks, which may reflect the relative maintenance of brain integration properties and global efficiency [10, 19–22]. Another hypothesis explains the hyperconnectivity as a direct response to functional network disconnection in a dynamic system such as the human brain [51].

Intrigued by the functional network rearrangement of the brain hubs, we then investigated the structural changes of the hubs themselves and the other brain regions occurring in aging. Our results find support in previous literature, reporting cortical thinning across the majority of the cortex between 20 and 85 years of age [4–7], and are consistent with the “last in, first out” hypothesis, according to which the prefrontal-inferior parietal-temporal brain areas, which are late-maturing regions, are preferentially the first to be vulnerable to aging [52, 53]. Indeed, we found that the highest cortical thinning is centered on FTP hubs and regions within fronto-temporo-parietal areas, and the slowest thinning is restricted to OCC hubs and sensorimotor and pre-motor cortex.

Investigating between-gender differences, men and women were characterized by decreased cortical thickness and functional connectivity in all the brain lobes during aging, with a greater involvement of the frontal and temporal lobe in men relative to women. These results are in line with previous findings suggesting a more severe structural and functional degeneration in frontal and temporal areas during aging in men [54]. Those differences might be attributed to a combination of genetic and hormonal factors that probably contribute to the increased vulnerability of certain brain regions [54].

Functional and structural alterations accumulating within brain hubs with aging may be a conduit for the spreading of neurodegeneration. Importantly, we found a relationship between the strength of functional connectivity with hubs in young subjects and the cortical thickness of connected regions in old subjects, suggesting a spatial similarity of age-related brain structural changes with the functional network maps of hubs. Specifically, a functional proximity to the FTP hubs was a risk factor for cortical thinning with aging, while an opposite pattern was observed for OCC hubs. As a further confirmation, cortical regions highly functionally connected to FTP hubs showed greater cortical thinning relative to those highly functionally connected to OCC hubs. It is becoming increasingly clear that brain network organization shapes the course and expression of neurodegenerative diseases [32, 55, 56]. Our findings reveal a significant role for hub connectome topology and geometry in shaping the accumulation of biological damage and distribution of atrophy with aging. Age-related neurodegeneration through hub connections could be due to common gene or protein expression signatures, loss of trophic factors, and/or transneuronal spread of toxic agents [57].

The study is not without limitations. Although the neuropsychological characterization of our sample was very comprehensive, there is a lack of information about lifestyle risk factors (i.e., smoking, obesity, lifestyle, health risk factors), which might modulate the brain aging changes and were not considered in the analyses. Another limitation lies in the cross-sectional nature of the study. Our findings may relate to the nature of the aging profiles, which represent snapshots in time. Longitudinal studies are warranted to verify the trajectories of changes. Finally, further investigations are warranted to evaluate the aging effect on subcortical areas.

In conclusion, our findings revealed potential patterns of vulnerability to aging, pointing out how functional network rearrangements of brain hubs and their structural change trajectories across lifespan influence the functional and structural trend of changes of the remaining brain regions.

### Supplementary information

Supplementary information
