# Supplementary material for: Age-related vulnerability of the human brain connectome
Source: Mol Psychiatry. 2023 Jul 6;28(12):5350–8. doi: 10.1038/s41380-023-02157-1 (PMC11041755; doi:10.1038/s41380-023-02157-1)
Supplement: Supplementary file 1 — Supplementary information [file 41380_2023_2157_MOESM1_ESM.docx]

**SUPPLEMENTARY INFORMATION**

**for**

**Age-related vulnerability of the human brain connectome**

Massimo Filippi,^1-5^ Camilla Cividini^1^, Silvia Basaia^1^, Edoardo G. Spinelli^1^, Veronica Castelnovo^1^, Michela Leocadi^1,5^, Elisa Canu^1^, Federica Agosta.^1,2,5^

^1^Neuroimaging Research Unit, Division of Neuroscience, ^2^Neurology Unit, ^3^Neurophysiology Service, and ^4^Neurorehabilitation Unit, IRCCS San Raffaele Scientific Institute, Milan, Italy; ^5^Vita-Salute San Raffaele University, Milan, Italy.

***Corresponding author**: Massimo Filippi, MD, FEAN, FAAN. Full Professor of Neurology, Vita-Salute San Raffaele University, Chair, Neurology Unit, Chair, Neurorehabilitation Unit, Director, Neurophysiology Service, Director, MS Center, Director, Neuroimaging Research Unit, Division of Neuroscience, IRCCS San Raffaele Scientific Institute, Via Olgettina, 60, 20132 Milan, Italy; telephone/fax: +390226433958 /+390226433031 email: [filippi.massimo@hsr.it](mailto:filippi.massimo@hsr.it)

**Table S1.** Sociodemographic data and comprehensive neuropsychological findings in young and older healthy adults.

| **Sociodemographic data** | | | | |
| --- | --- | --- | --- | --- |
|  |  | **Young healthy adults** | **Older healthy adults** | **p-value** |
|  | Age [years] | 25.44 ± 3.01  (20.48 – 31.69) | 62.82 ± 9.35  (36.82 – 84.59) | **<0.001** |
|  | Sex [M/W] | 27/23 | 22/56 | **0.01** |
|  | Education [years] | 15.56 ± 2.98  (8.00 – 24.00) | 12.04 ± 3.75  (5.00 – 20.00) | **<0.001** |
| **Neuropsychological data** | | | | |
| **Global cognition** | MMSE^1^ | 29.82 ± 0.39  (29.00 – 30.00) | 29.38 ± 0.82  (27.00 – 30.00) | **0.002** |
| **Memory** | Digit Span, forward^8^ | - | 5.96 ± 1.00  (4.00 – 8.00) | - |
|  | RAVLT^2^  [immediate recall] | 57.54 ± 6.74  (43.00 – 70.00) | 48.99 ± 7.37  (31.00 – 67.00) | **<0.001** |
|  | RAVLT [delayed recall]^2^ | 12.79 ± 1.80  (8.00 – 15.00) | 10.64 ± 2.44  (4.00 – 15.00) | **<0.001** |
|  | RAVLT [recognition]^2^ | 14.88 ± 0.44  (13.00 – 15.00) | 14.30 ± 1.38  (6.00 – 15.00) | **0.03** |
|  | RAVLT [false positives]^2^ | 0.62 ± 2.00  (0.00 – 13.00) | 0.68 ± 1.71  (0.00 – 13.00) | 0.77 |
|  | Spatial span, forward^8^ | - | 5.29 ± 1.10  (3.00 – 7.00) | - |
|  | Rey’s figure [delayed recall]^3^ | 23.19 ± 5.48  (1.00 – 33.00) | 15.00 ± 5.76  (6.50 – 23.00) | **<0.001** |
|  | Benson’s figure [delayed recall]^4^ | - | 11.12 ± 3.16  (4.00 – 17.00) | - |
|  | Benson’s figure [recognition]^4^ | - | 1.00 ± 0.00  (1.00 – 1.00) | - |
| **Attention and executive functions** | Trail Making Test (B-A)^5^ | 36.07 ± 14.47  (14.01 – 105.40) | 64.62 ± 38.03  (19.99 – 209.69) | **<0.001** |
|  | Digit Span, backward^11^ | - | 4.77 ± 1.24  (3.00 – 8.00) | - |
|  | Attentive matrices^9^ | - | 51.77 ±8.46  (4.00 – 60.00) | - |
|  | Phonemic fluency^12^ | - | 37.60 ± 8.18  (18.00 – 59.00) | - |
|  | Semantic fluency^12^ | - | 47.48 ± 9.26  (27.00 – 70.00) | - |
|  | Raven CPM^10^ | - | 32.00 ± 3.29  (17.00 – 36.00) | - |
|  | MCST, categories^6^ | 5.52 ± 0.88  (3.00 – 6.00) | 4.42 ± 1.34  (1.00 – 6.00) | **<0.001** |
|  | MCST, perseverations^6^ | 1.30 ± 2.32  (0.00 – 10.00) | 3.57 ± 3.51  (0.00 – 16.00) | **<0.001** |
|  | Pasat 2”^15^ | 40.83 ± 10.17  (18.00 – 58.00) | - | - |
| **Language** | Token Test^13^ | - | 34.14 ± 1.70  (28.00 – 36.00) | - |
|  | Naming to oral description [CaGi]^17^ | 47.13 ± 1.19  (43.00 – 48.00) | - | - |
| **Visuospatial** | Rey’s figure [copy]^3^ | 32.86 ± 2.12  (26.00 – 36.00) | 28.79 ± 3.75  (19.50 – 33.00) | **<0.001** |
|  | Benson’s figure [copy]^4^ | - | 15.72 ± 0.77  (14.00 – 17.00) | - |
|  | Copy of drawings [freehand]^2^ | - | 10.27 ± 1.63  (4.00 – 12. 00) | - |
|  | Copy of drawings with landmarks^2^ | - | 67.37 ± 3.60  (51.00 – 70.00) | - |
|  | Benton line orientation [30 lines]^16^ | 27.40 ± 2.61  (18.00 – 30.00) | - | - |
| **Mood**  **and**  **behavior** | BDI^7^ | 5.40 ± 4.68  (0.00 – 24.00) | 7.26 ± 4.94  (0.00 – 23.00) | 0.31 |
|  | Apathy Rating Scale^14^ | - | 6.33 ± 5.10  (0.00 – 19. 00) | - |
|  | SCL-90-R [Total]^18^ | 28.95 ± 30.64  (2.00 – 163.00) | - | - |

Values are reported as mean ± standard deviation (range). Differences in sociodemographic data were assessed using ANOVA models or Chi-squared test (p<0.05). On the other hand, differences in neuropsychological profile between young and older healthy adults were assessed using one-way ANOVA corrected for age, sex and education (p<0.05). P values were adjusted for Bonferroni- multiple comparison. *Abbreviations*: BDI= Beck Depression Inventory; CPM= coloured progressive matrices; MCST= Modified Card Sorting test; M= Men; MMSE= Mini Mental State Examination; RAVLT= Rey Auditory Verbal Learning Test; SCL-90-R=Symptom Checklist-90-Revised, W= Women.

**eREFERENCES**

1. Folstein MF, Folstein SE, McHugh PR. "Mini-mental state". A practical method for grading the cognitive state of patients for the clinician. *J Psychiatr Res*. 1975;12(3):189-98.

2. Carlesimo GA, Caltagirone C, Gainotti G. The Mental Deterioration Battery: normative data, diagnostic reliability and qualitative analyses of cognitive impairment. The Group for the Standardization of the Mental Deterioration Battery. *Eur Neurol*. 1996;36(6):378-84.

3. Caffarra P, Vezzadini G, Dieci F, Zonato F, Venneri A. Rey-Osterrieth complex figure: normative values in an Italian population sample. *Neurol Sci*. 2002;22(6):443-7.

4. Possin KL, Laluz VR, Alcantar OZ, Miller BL, Kramer JH. Distinct neuroanatomical substrates and cognitive mechanisms of figure copy performance in Alzheimer's disease and behavioral variant frontotemporal dementia. *Neuropsychologia*. 2011;49(1):43-8.

5. Giovagnoli AR, Del Pesce M, Mascheroni S, Simoncelli M, Laiacona M, Capitani E. Trail making test: normative values from 287 normal adult controls. *Ital J Neurol Sci*. 1996;17(4):305-9.

6. Caffarra P, Vezzadini G, Dieci F, Zonato F, Venneri A. Modified Card Sorting Test: normative data. *J Clin Exp Neuropsychol*. 2004;26(2):246-50.

7. Beck AT, Ward CH, Mendelson M, Mock J, Erbaugh J. An inventory for measuring depression. *Arch Gen Psychiatry*. 1961;4:561-71.

8. Orsini A, Grossi D, Capitani E, Laiacona M, Papagno C, Vallar G. Verbal and spatial immediate memory span: normative data from 1355 adults and 1112 children. *Ital J Neurol Sci*. 1987;8(6):539-48.

9. Tognoni HSaG. *Standardizzazione e Taratura Italiana di Test Neuropsicologici*. vol Vol. S8. The Italian Journal of Neurological Sciences. 1987:44-46.

10. Basso A, Capitani E, Laiacona M. Raven's coloured progressive matrices: normative values on 305 adult normal controls. *Funct Neurol*. 1987;2(2):189-94.

11. Monaco M, Costa A, Caltagirone C, Carlesimo GA. Forward and backward span for verbal and visuo-spatial data: standardization and normative data from an Italian adult population. *Neurol Sci*. 2013;34(5):749-54.

12. Novelli G, Laiacona M, Papagno C, Vallar G, Capitani E, Cappa SF. Three clinical tests to research and rate the lexical performance of normal subjects. *Arch Psicol Neurol Psichiatr* 1986;47(4):477-506.

13. De Renzi E, Vignolo LA. The token test: A sensitive test to detect receptive disturbances in aphasics. *Brain*. 1962;85:665-78.

14. Marin RS, Biedrzycki RC, Firinciogullari S. Reliability and validity of the Apathy Evaluation Scale. *Psychiatry Res*. 1991;38(2):143-62.

15. Amato MP, Portaccio E, Goretti B, et al. The Rao's Brief Repeatable Battery and Stroop Test: normative values with age, education and gender corrections in an Italian population. *Mult Scler*. 2006;12(6):787-93.

16. Benton AL, Varney NR, Hamsher KD. Visuospatial judgment. A clinical test. *Arch Neurol*. 1978;35(6):364-7.

17. Catricala E, Della Rosa PA, Ginex V, Mussetti Z, Plebani V, Cappa SF. An Italian battery for the assessment of semantic memory disorders. *Neurol Sci*. 2013;34(6):985-93.

18. Derogatis LR, Savitz KL. The SCL–90–R and Brief Symptom Inventory (BSI) in primary care. 2000;

**Table S2.** MRI acquisition parameters.

| **Milan** | Philips Medical System Ingenia CX 3T scan | | | | |
| --- | --- | --- | --- | --- | --- |
|  | 3D T2-weighted SE | 3D FLAIR | 3D T1-weighted TFE | Diffusion weighted sequence | T2*-weighted single-shot EPI sequence  (RS fMRI) |
| **Repetition time (msec)** | 2500 | 4800 | 7 | 5900 | 1567 |
| **Echo time (msec)** | 330 | 267 | 3.2 | 78 | 35 |
| **Flip angle** | - | 90° | 9° | - | 70° |
| **Section thickness (mm)** | 1 | 1 | 1 | 2.3 | 3 |
| **No. of sections** | 192 | 192 | 204 | 56 | 48 for 320 volumes |
| **Matrix** | 256x256 | 256x256 | 256x240 | 112x85 | - |
| **Field of view (mm^2^)** | 256x256 | 256x256 | 256x240 | 240x232 | 240x240 |
| **Diffusion gradient directions** | - | - | - | 6/30/60 | - |
| ***b* value sec/mm^2^** | - | - | - | 700/1000/2855 | - |

Abbreviations: FFE= fast field echo; FLAIR= fluid-attenuated inversion recovery; MRI= magnetic resonance imaging; msec= millisecond; mm= millimeter; No= number; RS fMRI= resting state functional MRI; SE=spin echo; sec=second.

**Table S3.** Between-group differences in stepwise functional connectivity maps.

| **Older Healthy Adults vs. Young Healthy Adults** | | | |
| --- | --- | --- | --- |
| **Seed of interest** | **Functional connectivity** | **Direct connectivity** *(Step 1)* | **Indirect connectivity** *(Step 2-3-4)* |
| *Middle frontal gyrus*  [Figure 3A] | ↓  [yellow - red] | Frontal areas: superior frontal gyri, right rostral middle frontal gyrus and medial orbitofrontal cortex.  Parietal areas: right inferior parietal and right supramarginal gyrus.  Temporal areas: right middle and inferior temporal gyri. | Frontal areas: superior frontal gyri, medial orbitofrontal cortex and right caudal anterior cingulate.  Parietal areas: right inferior parietal cortex.  Left insular cortex. |
|  | ↑  [blue - green] | Parietal areas: superior parietal cortex.  Temporal areas: superior temporal gyrus, fusiform, right entorhinal cortex, right parahippocampal gyrus and left hippocampus.  Occipital areas: lingual gyrus, left pericalcarine, left cuneus, and left lateral occipital cortex | Sensorimotor areas: precentral and postcentral gyri.  Parietal areas: superior parietal cortex and right supramarginal gyrus.  Temporal areas: right superior temporal gyrus, right fusiform and left hippocampus.  Occipital areas: lingual gyri, left pericalcarine and left cuneus. |
| *Rostral anterior cingulate*  [Figure 3B] | ↓  [yellow - red] | Frontal areas: superior frontal gyri, medial orbitofrontal cortex and rostral anterior cingulate cortex.  Temporal areas: left entorhinal cortex and right hippocampus.  right isthmus cingulate cortex | Frontal areas: superior frontal gyri, medial orbitofrontal cortex, rostral anterior cingulate and right caudal anterior cingulate.  Parietal areas: precuneus, right posterior cingulate and right isthmus cingulate.  Left insular cortex.  Temporal areas: left entorhinal cortex and right hippocampus. |
|  | ↑  [blue - green] | Frontal areas: caudal medial superior frontal gyri, left rostral middle frontal gyrus, left pars opercularis and right caudal anterior cingulate.  Sensorimotor areas: postcentral gyri,  Parietal areas: superior parietal cortex and supramarginal gyri) | Temporal areas: right fusiform gyrus  Occipital areas: right lingual gyrus and right lateral occipital cortex. |
| *Precuneus*  [Figure 3C] | ↓  [yellow - red] | Frontal areas: superior frontal gyri, medial orbitofrontal cortex, rostral anterior cingulate.  Parietal areas: precuneus, isthmus cingulate and posterior cingulate.  left insula  Occipital areas: pericalcarine and cuneus. | Parietal areas: right inferior parietal cortex.  Temporal areas: left inferior temporal gyrus. |
|  | ↑  [blue - green] | Sensorimotor areas: precentral and postcentral gyri.  Parietal areas: superior parietal cortex and supramarginal gyri.  Temporal areas: superior temporal gyri and right fusiform.  Occipital areas: right lateral occipital cortex. | Occipital areas: lateral occipital cortex, lingual gyri, left pericalcarine, left cuneus. |
| *Posterior cingulate*  [Figure 3D] | ↓  [yellow - red] | Frontal areas: right superior frontal gyrus, medial orbitofrontal cortex and caudal anterior cingulate.  Right insular cortex. | Frontal areas: superior frontal gyri, medial orbitofrontal cortex and right caudal anterior cingulate.  Insular cortex.  Temporal areas: left transverse temporal cortex. |
|  | ↑  [blue - green] | Sensorimotor areas: precentral and postcentral gyri.  Parietal areas: superior parietal cortex.  Temporal areas: left middle temporal gyrus. | Sensorimotor areas: precentral and postcentral gyri.  Parietal areas: superior parietal cortex.  Temporal areas: Right fusiform gyrus.  Occipital areas: lingual gyri, pericalcarine and cuneus. |
| *Inferior Parietal cortex*  [Figure 3E] | ↓  [yellow - red] | Frontal areas: rostral middle frontal gyri, medial orbitofrontal cortex and pars orbitalis.  Parietal areas: right inferior parietal lobule.  Left insular cortex. | Frontal areas: superior frontal gyri, medial orbitofrontal cortex and right caudal anterior cingulate.  Insular cortex. |
|  | ↑  [blue - green] | Sensorimotor areas: precentral and postcentral gyri.  Parietal areas: superior parietal cortex.  Temporal areas: right fusiform gyrus and right parahippocampal gyrus.  Occipital areas: lingual gyrus. | Parietal areas: superior parietal cortex and right supramarginal.  Temporal areas: right superior temporal gyrus and right fusiform gyrus.  Occipital areas: right lingual gyrus, left pericalcarine and left cuneus. |
| *Middle temporal gyrus*  [Figure 3F] | ↓  [yellow - red] | Frontal areas: superior frontal gyri and medial orbitofrontal cortex.  Parietal areas: right inferior parietal cortex.  Temporal areas: middle temporal and inferior temporal gyri. | Frontal areas: right caudal anterior cingulate.  Left insular cortex.  Parietal areas: right posterior cingulate. |
|  | ↑  [blue - green] | Frontal areas: left rostral middle frontal gyrus and left paracentral lobule.  Parietal areas: superior parietal gyri, supramarginal gyri.  Right insular cortex.  Temporal areas: right transverse temporal gyrus, superior temporal gyri.  Occipital areas: lateral occipital cortex | Sensorimotor areas: precentral and postcentral gyri.  Parietal areas: superior parietal gyrus and right supramarginal gyrus.  Temporal areas: superior temporal gyri.  Occipital areas: lingual gyrus, left pericalcarine and left cuneus. |
| *Lingual gyrus*  [Figure 3G] | ↓  [yellow - red] | Frontal areas: superior frontal gyri and medial orbitofrontal cortex.  isthumus cingulate cortex  Occipital areas: lingual gyri. | Frontal areas: superior frontal gyri, medial orbitofrontal cortex and right caudal anterior cingulate.  Parietal areas: precuneus and right posterior cingulate.  Left insular cortex. |
|  | ↑  [blue - green] | Frontal areas: right caudal middle frontal gyrus.  Sensorimotor areas: precentral and postcentral gyri.  Parietal areas: superior parietal cortex and right supramarginal gyrus.  Insular cortex.  Temporal areas: right superior temporal gyrus and inferior temporal gyri. | Temporal areas: right fusiform gyrus  Occipital areas: lingual gyri, pericalcarine and cuneus. |
| *Pericalcarine cortex*  [Figure 3H] | ↓  [yellow - red] | Frontal areas: superior frontal gyri, medial orbitofrontal cortex and rostral anterior cingulate.  Insular cortex.  Temporal areas: left transverse temporal gyrus, isthmus cingulate cortex.  Occipital areas: lingual gyri and cuneus. | Frontal areas: superior frontal gyri, medial orbitofrontal cortex, left rostral anterior cingulate and right caudal anterior cingulate.  Parietal areas: precuneus and posterior cingulate.  Left insular cortex. |
|  | ↑  [blue - green] | Sensorimotor areas: precentral and postcentral gyri.  Parietal areas: superior parietal cortex and right supramarginal gyrus.  Temporal areas: right superior temporal gyrus.  Occipital areas: right lateral occipital cortex) | Sensorimotor areas: precentral and postcentral gyri.  Parietal areas: superior parietal cortex and right supramarginal gyrus. Temporal areas: right superior temporal gyrus and right fusiform gyrus.  Occipital areas: lingual gyri, pericalcarine, cuneus and lateral occipital cortex. |

**
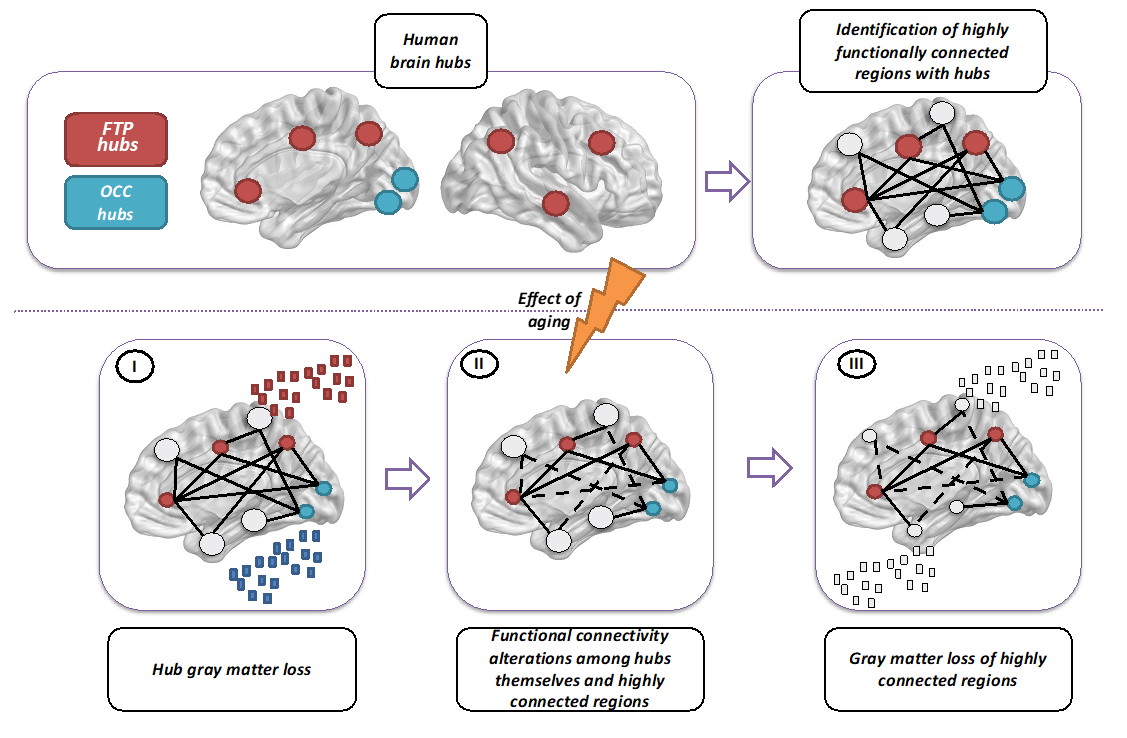
**

**Figure S1. Study hypothesis.** Selection of brain hubs as pivotal regions of the functional brain network (Top row). **I.** Characterization of stepwise functional connectivity patterns of each hub to identify regions highly functionally connected to hubs. **II.** Hub grey matter loss: evaluation of cortical thickness changes across lifespan for selected hubs and the remaining brain regions. **III.** Evaluation of stepwise functional connectivity alterations among hubs and highly connected regions with aging **IV.** The effect of functional vulnerability and atrophy of brain hubs on highly connected regions.


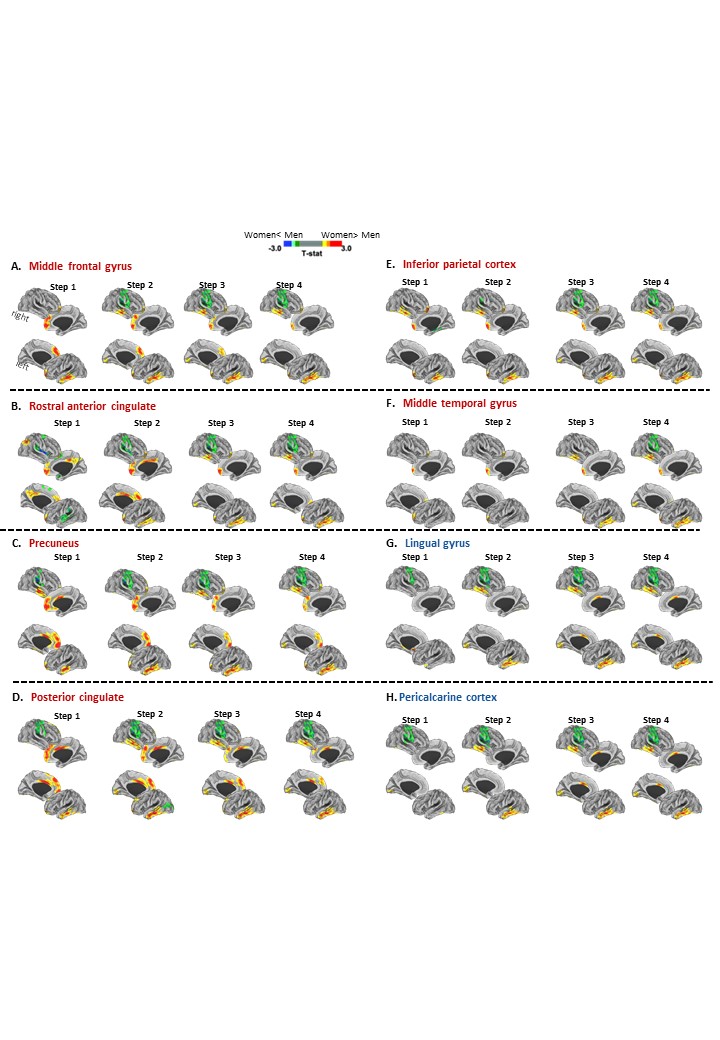


**Figure S2.** **Differences between men and women in old healthy adults in stepwise functional connectivity of the eight hubs.** Cortical maps represent the significant differences in stepwise functional connectivity values between men and women healthy adults. Statistical analysis was adjusted for education. Results were corrected for multiple comparisons using a threshold-free cluster enhancement method combined with nonparametric permutation testing at p<0.05 FWE-corrected. Color bars show the t-statistic applicable to the image. Red-yellow areas represent decreased functional connectivity in men relative to women, whereas blue/green areas represent enhanced functional connectivity in men relative to women.


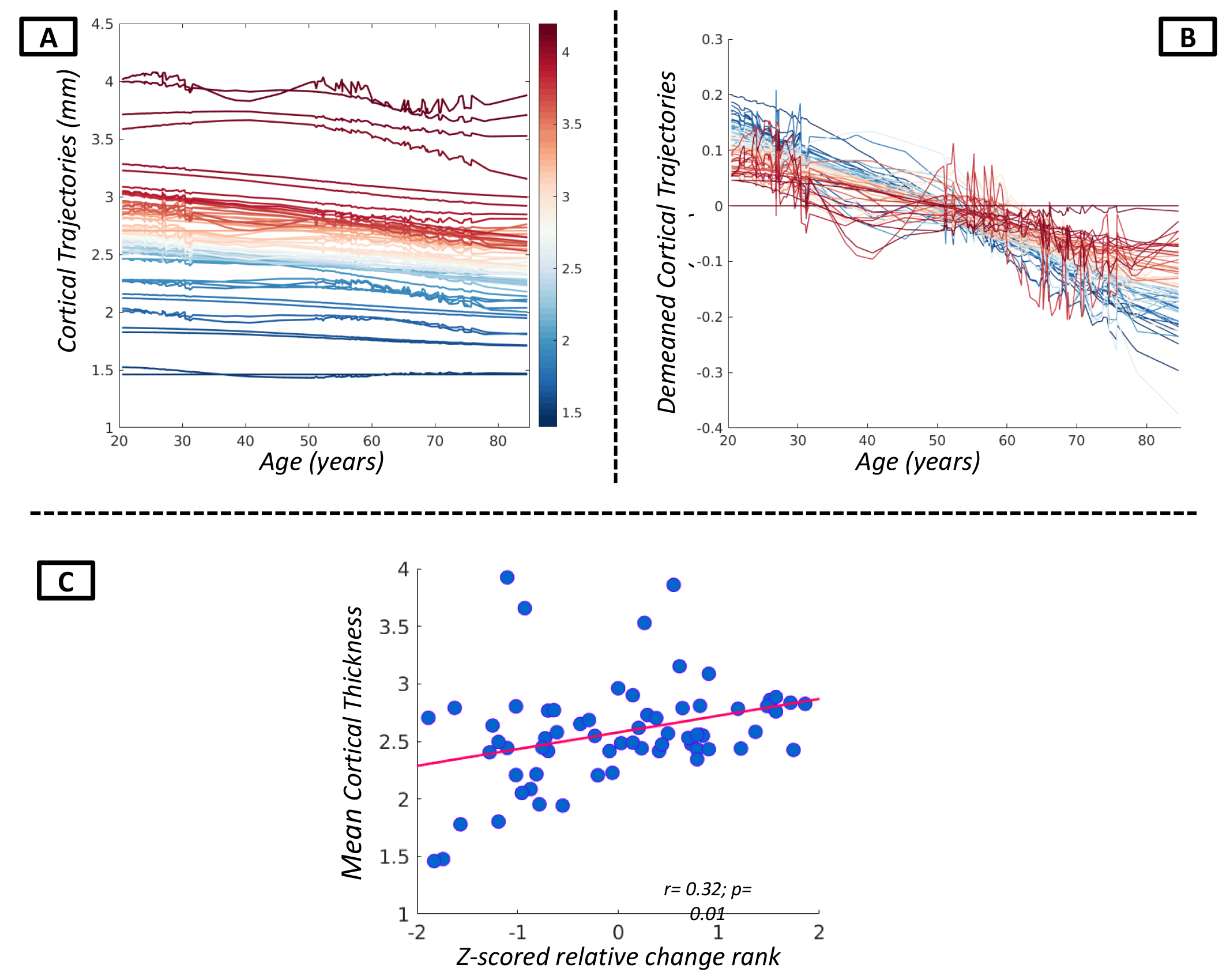


**Figure S3. Modelling of cortical thickness trajectories across lifespan. A**. The estimated trajectories of cortical thickness value for each cortical region in the observed timeframe are reported, colored by mean cortical thickness value (red= the highest value, blue= the lowest value). **B.** The demeaned estimated cortical trajectories for each region are reported (blue = greater change). **C.** Correlation between regional mean cortical thickness and the relative change with age are reported.


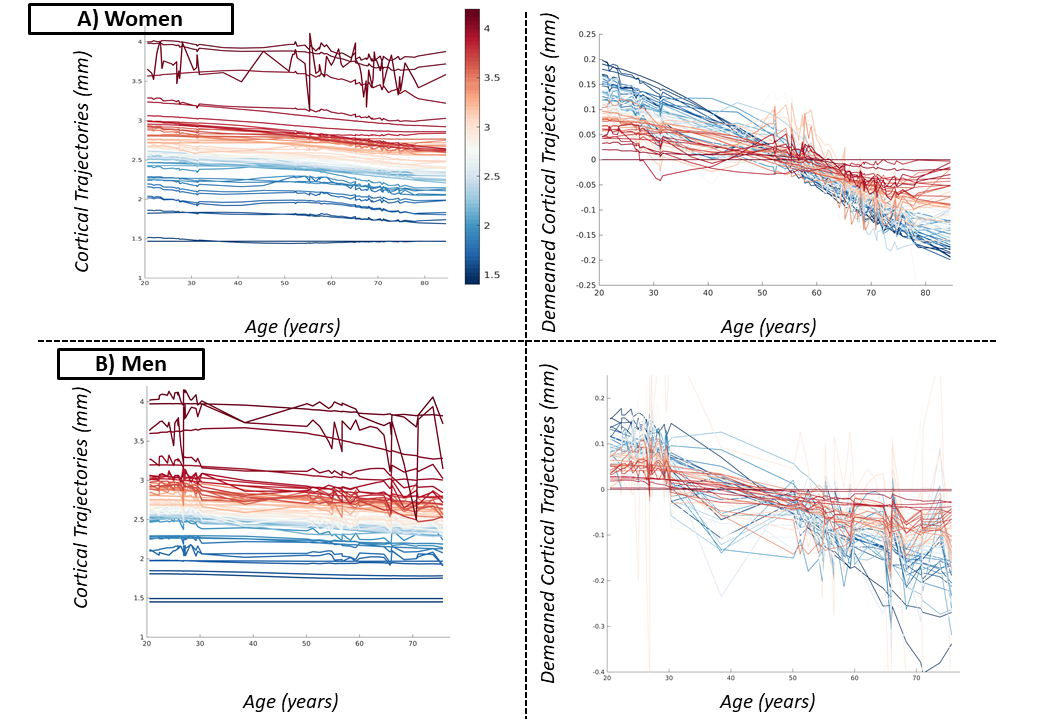


**Figure S4. Modelling of cortical thickness trajectories across lifespan in men and women. A**. The estimated trajectories of cortical thickness value for each cortical region (red= the highest value, blue= the lowest value) in the observed timeframe and the demeaned estimated cortical trajectories for each region (blue = greater change) are reported in women, colored by mean cortical thickness value. **B.** The estimated trajectories of cortical thickness value for each cortical region (red= the highest value, blue= the lowest value) in the observed timeframe and the demeaned estimated cortical trajectories for each region (blue = greater change) are reported in men, colored by mean cortical thickness value.
